# Mediating Impact of Intranasal Oxytocin on the Interaction Between Irritability and Reactive Aggression in Youth with Severe Irritability

**DOI:** 10.3390/life15081253

**Published:** 2025-08-07

**Authors:** Jake J. Son, Ji-Woo Suk, William F. Garvey, Ryan T. Edwards, Ellen Leibenluft, R. J. R. Blair, Soonjo Hwang

**Affiliations:** 1 College of Medicine, University of Nebraska Medical Center, Omaha, NE 68198, USA; jake.son@unmc.edu; 2 Korean Institute of Alternative Medicine, Daejon 34054, Republic of Korea; suk6124@naver.com; 3 School of Psychology, University of Sheffield, Sheffield S10 2TN, UK; willgarvey82@gmail.com; 4 Department of Psychiatry, University of Nebraska Medical Center, Omaha, NE 68198-5578, USA; ryan.edwards@unmc.edu; 5 Section on Mood Dysregulation and Neuroscience, National Institute of Mental Health, Bethesda, MD 20892, USA; leibs@mail.nih.gov; 6 Child and Adolescent Mental Health Centre, Mental Health Services, Capital Region of Denmark, 2900 Copenhagen, Denmark; robert.james.blair@regionh.dk

**Keywords:** irritability, reactive aggression, oxytocin, superior prefrontal cortex

## Abstract

Objective: Irritability and reactive aggression are transdiagnostic features that are predictive of adverse long-term outcomes. This investigation examined whether intranasal oxytocin administration impacts the interaction between irritability and reactive aggression, and whether these effects can be detected at a neural level via a facial expression processing task during functional MRI (fMRI). Methods: In this study, 40 children and adolescents with severe irritability and psychiatric diagnoses of disruptive mood and behavioral disorders were assigned to either intranasal oxytocin or placebo administration over a 3-week period in a randomized, double-blind trial (ClinicalTrials, NCT02824627). Clinical measures and fMRI during a facial expression processing task were collected pre- and post-intervention. Brain regions sensitive to oxytocin administration were determined using whole-brain statistical analyses, with post hoc analyses to determine whether changes in the neural activity mediated the relationship between changes in irritability and reactive aggression across the intervention period. Results: Youth who received intranasal oxytocin administration exhibited significant decreases in irritability and reactive aggression compared to their counterparts in the placebo group. Further, oxytocin administration was associated with significant increases in neural activity in the right superior prefrontal cortex, which fully mediated the relationship between improvements in irritability and improvements in reactive aggression. Conclusions: Intranasal oxytocin significantly reduced irritability and reactive aggression in youth, as well as neural activity in the prefrontal cortex, such that increases in the cortical activity fully mediated the relationship between changes in irritability and reactive aggression. Taken together, these findings may reflect oxytocin-related enhancements in emotional regulation in youth with severe irritability, a potential therapeutic mechanism for mitigating reactive aggression.

## 1. Introduction

Childhood and adolescence are critical periods for the development of psychopathology and early intervention is key to mitigating long-term consequences [1,2]. Irritability is a common, transdiagnostic psychopathology in various mental health disorders of youth, including Attention-Deficit/Hyperactivity Disorder (ADHD), Oppositional Defiant Disorder (ODD), Conduct Disorder (CD), and Disruptive Mood Dysregulation Disorder (DMDD) [3]. Irritability has consistently been associated with an increased propensity for poor frustration tolerance, heightened emotional reactivity, and deficits in emotional regulation [4]. Critically, many of these symptoms persist well into adulthood, contributing to an increased likelihood of developing mental health disorders, interpersonal challenges, and substance use [5]. Specifically, irritability has been highly correlated with aggressive behavior, particularly reactive aggression, or impulsive, emotionally driven responses to perceived or real threats and frustrations [6].

The theoretical framework that aims to differentiate proactive and reactive forms of aggression is of great interest in their relation to irritability, given that the behavioral manifestations such as aggressive behavior are often the main reasons for clinical assessment and treatment for youth with severe levels of irritability [7,8]. Although these are dimensional processes that tend to co-occur [9], the main differentiating factor is the function served by proactive vs. reactive forms of aggression, such that proactive aggression is an intentional, premeditated act to achieve a specific goal [10], while reactive aggression is in response to stressors and/or threat cues in a self-protective or retaliatory fashion [11]. Thus reactive aggression can be considered conceptually and empirically as more closely related to irritability and behavioral endpoints [12]. From a clinical perspective, addressing irritability may result in a specific improvement in reactive aggression and associated behaviors.

Although both irritability and aggressive behavior have clinical significance in youth, there are very few studies targeting their interaction for treatment/intervention [13]. Recent evidence suggests that oxytocin plays a critical role in modulating emotional states of negative valence, as well as social and behavioral responses, particularly in youth with affective dysregulation [14,15,16,17]. Oxytocin is an oligopeptide hormone formed in the hypothalamus and released by the posterior pituitary that plays a critical role in reproduction and social behaviors [18]. Oxytocin modulates intracellular signaling as well as transcriptional changes including neurotransmitter production and synaptic plasticity that support long-term modifications to behavior [19]. These effects may be particularly impactful during development in the midst of significant changes in neurobiology, the quality and quantity of social interactions, and the adoption of adult-like responsibilities [20]. However, there is a relative dearth of the literature examining specific neural changes induced by oxytocin administration in response to facial expression processing, a critical skill for navigating an increasingly complex social and motivational landscape throughout development [21]. Numerous previous studies have demonstrated aberrant neural activation as the participants process facial expression in adults and youth with irritability and reactive aggression [6,22]. Further, previous studies of intranasal oxytocin implemented for various psychiatric diagnoses showed decreased activation in cortical regions implicated in emotional responding to negative stimuli after oxytocin administration [23]. More specifically, the prefrontal cortex may be particularly sensitive to oxytocin administration, given that these regions undergo protracted development, exhibit some of the highest measures of functional inter-individual variability, and play a critical role in emotion regulation [24,25,26]. Furthermore, the prefrontal cortex exhibited increased messenger RNA (mRNA) concentrations for the oxytocin receptor in post-mortem studies of patients with various psychiatric disorders, which may reflect the upregulation of receptors due to an insufficiency or impaired oxytocin signaling [27]. Thus, oxytocin appears to be an excellent candidate to examine the interaction between irritability and reactive aggression and begin to elucidate the neural mechanisms supporting such changes. While other pharmacologic interventions (e.g., selective serotonin reuptake inhibitors, dopamine precursors) have been utilized for the clinical trials on irritability in youth [28,29], the mechanisms supporting irritability are still not fully understood. The mechanisms underlying irritability may be uniquely modulated by oxytocin given its critical role in social behaviors that are widely implicated across a wide range of mental health disorders [19]. However, no studies to our knowledge have investigated the impact of oxytocin in youth with severe levels of irritability in the context of various psychiatric diagnoses (i.e., disruptive mood and behavior dysregulation disorders) on the neural activity targeting the interaction between irritability and reactive aggression, and whether these neural changes directly mediate behavioral improvements.

In this study, we analyzed a sample of 40 children and adolescents with severe levels of irritability in a double-blinded clinical trial of intranasal oxytocin administration, especially on the interaction between irritability and reactive aggression. Clinical measurements and functional MRI (fMRI) during a facial expression processing task were obtained prior to and shortly after a 3-week intervention period of intranasal oxytocin administration. Our goals were to elucidate region-specific alterations in the neural mechanisms by examining facial expression processing that may have mediated the interaction between irritability and reactive aggression.

## 2. Methods

### 2.1. Participants

This study was conducted as a part of a clinical trial of intranasal oxytocin for youth with severe levels of irritability. For the details of the clinical trial, please see Hwang et al., 2024 [30]. Full trial information is available online (https://clinicaltrials.gov/study/NCT02824627, accessed on 27 January 2017). This study is a parallel assignment intervention. We obtained approval from the Institutional Review Board of the University of Nebraska Medical Center. Consent and assent were obtained from all participants and their parents/legal guardians.

### 2.2. Screening/Assessment

Psychiatric diagnoses were confirmed by the semi-structured Kiddie-Schedule for Affective Disorders and Schizophrenia for School-Age Children-Present and Lifetime Version (K-SADS: PL) [31]. Inclusion criteria were as follows: (1) aged 10–18 years, (2) a current diagnosis of ADHD, ODD, CD, or DMDD as determined by the K-SADS, (3) a clinically significant level of irritability as defined by a score of 4 or greater (≥4) either on the self-reported or parent-reported Affective Reactivity Index (ARI) [32], and (4) if currently on psychotropic medication, treatment must be unchanged for at least 6 weeks (medications participants were taking at the time of study included stimulant medication, alpha 2 agonist, atomoxetine, antipsychotics, mood stabilizers, or antidepressants) prior to enrollment. Exclusion criteria were as follows: (1) comorbid psychotic, tic (due to potential excessive movement under MRI scan), or substance use disorders, (2) major medical illness that prohibits treatment with oxytocin (e.g., severe liver disease, seizure disorder, or metabolic disorder), (3) past history of allergic reaction to oxytocin and its nasal spray product, (4) history of CNS disease (including history of seizure, epilepsy, CNS tumor, CNS hemorrhage, or serious CNS infection including meningitis or encephalitis), (5) current use of anxiolytics (benzodiazepines and barbiturates), (6) a positive urine pregnancy test, (7) a positive urine drug screen or any history of diagnosis of a substance use disorder, (8) Wechsler Abbreviated Scale of Intelligence, Second Edition (WASI-II) scores < 70 [33], (9) metal in the body (i.e., hearing aid, cardiac pacemaker, bone plates, braces, non-removable piercings/implants, etc.), claustrophobia, or any other contraindication to fMRI scanning, and (10) primary diagnosis of autism spectrum disorder (ASD) with associated impairments in communication and significant behavioral disturbance (at the initial chart review, if the primary reason for psychiatric referral was pharmacological intervention for severe ASD, the patient was not approached for recruitment).

The following symptom profiles were assessed: (1) clinical severity of irritability by the Affective Reactivity Index (ARI; 7-item inventory, self- and parent-report versions) to quantify irritability) and (2) severity of aggressive behavior by the Reactive-Proactive Aggression Questionnaire (RPAQ), a 23-item inventory to quantify aggressive behavior (reactive [RA] and proactive aggression [PA]) [34]. These measures were completed twice before and after the intervention, on average, 3 weeks apart.

### 2.3. Double-Blind, Randomized Intranasal Oxytocin Administration

At the academic institution where this study was conducted, the investigational pharmacist provided a parallel assignment randomization process via a computer-generated sequence of oxytocin (OXT) or placebo (PBO) administration. All research personnel were blinded to the assignment of participants until the conclusion of this study. For 3 weeks, participants either received 24 IU of intranasal oxytocin (OXT) daily, or in some cases, 12 IU if his/her weight was below 40 kg (n = 3), or placebo (PBO). Under the supervision of legal guardians, the participants self-administered the study drug based on their assigned group using the bottle provided at the first study visit. All participants were instructed to administer the medication in the morning, though they were permitted to move the administration time to night in cases of daytime drowsiness. A follow-up visit was scheduled at week 2, to assess participants’ compliance, any potential adverse events (AEs), and other clinical issues. We also instructed participants to bring the bottle to the final visit to ensure the participant has been compliant with daily administration, by assessing the remaining amount of the solution (either oxytocin or placebo). Clinically appropriate routine psychiatric care was allowed throughout the study participation. Participants were excluded if there was any change in their psychiatric medications during the study participation.

### 2.4. Functional MRI Experimental Design

**Figure 1:**
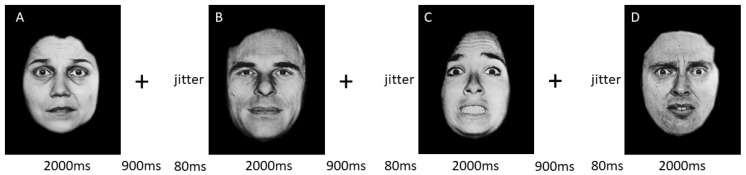
Facial expression task.

The facial expression task in this study was modified from previous work, see Figure 1 [35]. All participants were shown images of 10 men and women from the Pictures of Facial Affect series [36]. These images exhibited various facial expressions with parametrically modulated intensities (50%, 100%, and 150%) for fearful expressions. Modulation was completed by morphing neutral and 100% fearful expressions to create composites (50% intensity) or by extrapolating from emotional expressions to create exaggerated expressions (150% intensity). Since neutral expressions may appear threatening [37], we utilized neutral and happy expressions to create facial expressions of 25% happiness, which can be seen as neutral [38]. During the scan, the faces were presented in a randomized fashion for all participants. Participants were asked to determine the gender of the face presented on the screen using two response buttons while implicitly processing the emotional valence and intensity. These methods have been found to enhance blood oxygen level-dependent (BOLD) responses to emotional stimuli [39]. Stimuli were projected onto a mirror in the MRI scanner from a computer display. Stimulus presentations occurred in one run (duration = 5 min and 50 s), consisting of 160 events (80 face trials, with 20 trials for each intensity) in a random order, where each trial included a face (duration: 2 s) followed by a fixation cross (duration: 900 milliseconds) and 80 interspersed “jittered” trials. These runs were preceded and concluded by four and five fixation trials, respectively (duration: 3 s).

### 2.5. Functional MRI Scans

Participants were invited to complete their initial MRI session after the initial assessment session and before the start of intranasal OXT or PBO. The average length between the 1st MRI scan and initiation of treatment was 1.3 days (SD = 0.62). They were invited back for the 2nd MRI session after the final post-intervention assessment session (3 weeks later). The average length between the end of treatment and the second MRI scan was 2.3 days (SD = 0.64). The MRI technicians and research staff who conducted the MRI procedure also remained blinded to the randomization status (OXT vs. PBO) of the participants. After completion of this study, routine psychiatric treatment continued at the outpatient clinic or by their local providers.

### 2.6. Image Acquisition

We used a 3.0 Tesla Siemens Prisma scanner with 32-channel head coils. The functional T2* weighted images were obtained by an echo-planar single-shot gradient t echo pulse sequence after sagittal localization (repetition time [TR] = 2500 ms, echo time [TE] = 27 ms, flip angle 90°, field-of-view (FOV) = 240 mm, 94 × 94 matrix, and 2.6 × 2.6 × 2.5 mm voxels). Image acquisition was conducted in 46 slices of 2.5 mm per brain volume (distance factor 21%). The duration of each run was 8 min and 16 s. A high-resolution T1-weighted anatomical image was obtained to support spatial normalization (TR = 2200 ms, TE = 2.48 ms, flip angle = 8°; FOV = 230 mm (0.9 × 0.9 × 0.1 mm^3^ voxels), 176 axial slices, 256 × 208 matrix, thickness = 1.0 mm, and distance factor 50%) registering the echo-planar imaging (EPI) data set covering the whole brain.

### 2.7. Imaging Data Preprocessing

The fMRI data were preprocessed and analyzed with the Analysis of Functional NeuroImages (AFNI) software package, version 23.12.04. [40] We discarded the first four repetitions of functional images collected before equilibrium magnetization at the individual level. We then registered the participants’ anatomical scans to the Talairach and Tournoux atlas individually [41]. The functional EPI data of each individual were then registered to their Talairach anatomical scan. To reduce variability among individuals and generate group maps, the EPI datasets for each participant were spatially smoothed (isotropic 6 mm^3^ Gaussian kernel). We normalized the time series data by dividing the signal intensity of a voxel at each time point by the mean signal intensity of that voxel for each run and multiplying the result by 100. This produced regression coefficients represent percent-signal change. We also censored every TR on which motion exceeded 1 mm.

For individual-level processing, a model was generated with six motion regressors and the following three regressors: (1) facial expression, (2) facial expression with weighting based on emotion intensity, and (3) incorrect responses. We performed GLM fitting with these three regressors as well as six motion regressors and a regressor modeling a first-order baseline drift function. We convolved all regressors with the hemodynamic response function (HRF) to account for the slow hemodynamic response, resulting in a β coefficient and an associated *t* statistic, for each voxel and regressor. No significant regressor collinearity was identified. Participants with >10% of TRs were defined as individuals with excessive movement and were discarded (movement above study limits (1.0 cm)). As such, data from five participants were not included in the final data analyses. Three ANOVAs (participants who received OXT vs. participants who received PBO) were conducted on the motion regressors. These analyses showed that there was no significant group difference in the movement parameters for the participants included in the final analyses; [F(1,39) = 1.63–2.32, *p* > 0.5].

Two participants who received intranasal OXT and three participants who received PBO received only one scan due to various reasons, including scheduling conflicts, machine breakdown, or withdrawal before the second scan. This resulted in twenty-two participants who received intranasal OXT and nineteen treated with PBO who completed 2 fMRI scans (pre- and post-treatment). Among those, one participant with intranasal OXT were excluded due to excessive movement. Thus, we included the data from 21 participants in the OXT group and 19 in the PBO group (total of 40 participants) in the final MRI data analyses.

### 2.8. Statistical Analysis

#### 2.8.1. Statistical Analysis of Clinical Data

The symptom profiles (irritability in ARI, proactive, and reactive aggressive behavior in RPAQ) at baseline between the two groups (OXT vs. PBO) were examined by a *t*-test. A 2 (group: OXT treatment and PBO) by 2 (time: pre- and post-treatment) repeated measure analysis of covariance analyses of variance (ANCOVA) on the symptom profiles was conducted to examine the change/improvement of the symptoms between these two groups (OXT vs. PBO).

#### 2.8.2. Behavioral Data

Two full 2 (group: OXT treatment and PBO) by 2 (time: pre- and post-treatment) repeated measured analysis of covariance (ANCOVA) were conducted on the accuracy and reaction time data with sex as covariates.

#### 2.8.3. MRI Data

Two approaches were selected to examine the BOLD response changes: First, a 2 (groups (between subject variable): OXT treatment and PBO) by 2 (times (within subject variable): pre- and follow-up) ANCOVA on the whole brain BOLD response data via 3 dMVM with sex as covariates was conducted. For this, we performed a correction for multiple comparisons using a spatial clustering operation in AFNI’s 3 dClustSim utilizing the auto-correction function (-acf) with 10,000 Monte Carlo simulations for a whole-brain grey matter mask. We set the initial threshold at *p* = 0.001 [42]. This procedure yielded an extent threshold of *k* = 25 voxels, resulting in a cluster-level false-positive probability of *p* < 0.05, corrected for multiple comparisons. We also provided effect sizes (Partial η^2^) for all clusters for the future meta-analysis.

### 2.9. Mediation Analysis

We conducted a mediation analysis to examine whether the differential blood oxygen level-dependent (BOLD) responses in the area showing significant group-by-time interactions (in this case, the superior frontal gyrus) mediated the association between the degree of changes in irritability and aggressive behavior. To do this, we extracted the beta values from the superior frontal gyrus based on the fear vs. happiness contrast image for each participant. The hypothesis was that the association between the degree of improvement in irritability and aggressive behavior (especially reactive aggression) would be mediated by the degree of BOLD response changes in the superior frontal gyrus. Mediation analysis was conducted using the Sobel test accompanied by a bootstrapping method with N = 5000 bootstrap samples using the PROCESS macro procedure for SPSS version 26 (IBM). The model was estimated through Process Model 4. PROCESS reports bias-corrected 95% confidence intervals as indicators of significance.

## 3. Results

### 3.1. Clinical Characteristics

A summary of the participants’ demographic and clinical features is shown in Table 1. No participants required changes of their psychiatric medications or a higher level of care (such as inpatient psychiatric hospitalization) during this study. There was no significant difference in age, IQ, or gender distribution between the OXT and PBO groups.

### 3.2. Symptom Profile Changes

The symptom profiles and their changes are summarized in Table 2. There were no significant differences between symptom profiles at the baseline between the OXT and PBO groups by the paired *t*-test [*t* = 0.24–1.23, *p* = 0.11–0.49]. Repeated-measure ANCOVAs showed that there was a significant group-by-time interaction in self-reported irritability by ARI [F(1,38) = 21.70 *p* < 0.001], self-reported aggressive behavior [F(1,38) = 8.17, *p* = 0.007], as well as self-reported reactive aggression [F(1,38) = 8.90, *p* = 0.005]. There was no significant difference in self-reported proactive aggression between the two groups [F(1,38) = 0.38, *p* = 0.54]. There was no statistical significance between the two groups in parent-reported irritability (ARI-P) [F(1,38) = 1.69, *p* = 0.20], parent-reported aggressive behavior (RPAQ-P) [F(1,38) = 1.64, *p* = 0.21], and parent-reported proactive/reactive aggression (PA-P and RA-P) [F(1,38) = 2.70, *p* = 0.11 & F(1,38) = 0.73, *p* = 0.43, respectively].

### 3.3. Behavioral Data

Both groups successfully performed the task during the two fMRI sessions (average accuracy 95.3% (SD = 4.8), mean reaction time = 905.79 ms (SD = 108.02)). There was no significant effects of the group or group-by-time interactions for the accuracy and reaction time [F(1,38) = 0.12–2.11, *p* = 0.16–0.72]. No main effect of time for accuracy was observed [F(1,38) = 1.47, *p* = 0.24], though there was a main effect of time for RT [F(1,38) = 6.34, *p* = 0.02]. The participants were faster (899.29 (SD = 104.47) after the treatment compared to pre-treatment (915.97, (SD = 113.12)).

### 3.4. MRI Data

#### 3.4.1. Whole-Brain Analysis

**Figure 2:**
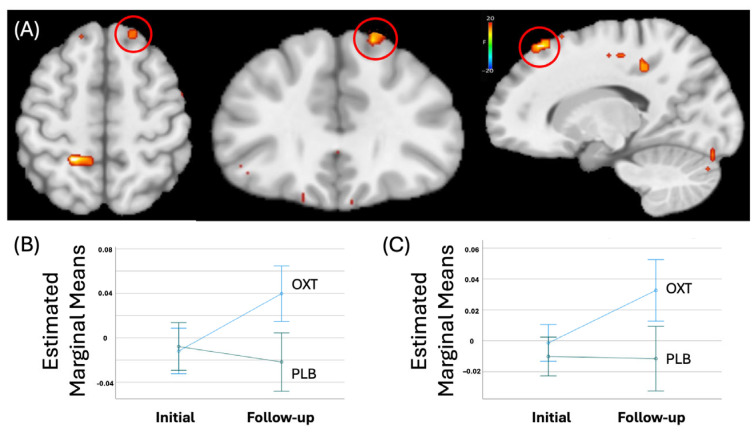
(**A**) Right superior frontal gyrus (coordinates:). In this area, the modulated BOLD responses to negative facial expression (**B**) and positive facial expression (**C**) showed significant group-by-time interaction. Youth with significant levels of irritability who received intranasal oxytocin (OXT) showed significant increase in the BOLD responses after the treatment, compared to youth with significant levels of irritability who received placebo (PBO).

Regions showing significant Group-by-Time interactions included the right superior frontal gyrus, see Table 3. Within this region, participants receiving OXT showed a significantly greater increase in activation in response to modulated negative [*t* = 5.00, *p* < 0.001] and positive facial expressions [*t* = 3.43, *p* < 0.001] after treatment relative to those receiving PBO, see Figure 2.

#### 3.4.2. Mediation Analysis

**Figure 3:**
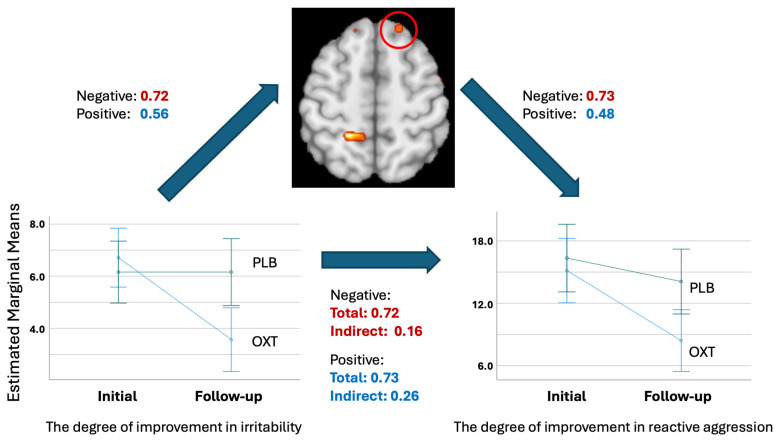
Mediation analysis model. Standardized beta coefficients (red: negative facial expressions; blue: positive facial expressions) are presented.

The mediation analysis demonstrated that the association between the degree of improvement in irritability and aggressive behavior was fully mediated through the degree of changes of the BOLD responses to modulated negative and positive facial expressions in the right superior frontal gyrus, see Figure 3.

First, the degree of improvement in irritability was regressed on the changes in modulated BOLD responses to negative and positive facial expressions. The standardized coefficients (β) were 0.72 (*p* < 0.001) for negative valence facial expressions and 0.56 (*p* < 0.001) for negative valence facial expressions, indicating that the degree of improvement in irritability strongly predicted the degree of changes in the BOLD responses. Similarly, the changes in BOLD responses to negative and positive facial expressions were regressed on the degree of improvement in reactive aggression. The standardized coefficients (β) were 0.73 (*p* = 0.00) and 0.48 (*p* = 0.005), respectively. Next, bootstrapping was performed to determine the indirect effect of the degree of improvement in irritability on the degree of improvement in reactive aggression. The total effect (β) was 0.72 (*p* = 0.006) and 0.72 (*p* = 0.006) for negative and positive facial expressions, and the indirect effect via mediator (ab) was 0.51 (CI = 0.42–1.55, *p* = 0.006) and 0.45 (CI = 0.13–1.58, *p* = 0.006), respectively (β), whereas the direct effects of improvement in irritability on improvement in reactive aggression was not statistically significant (0.16, CI = −0.76–0.45, *p* = 0.61; 0.26, *p* = 0.33, CI = −0.28–0.82, respectively). Thus, the indirect effect of the BOLD response changes in the superior prefrontal cortex to negative and positive facial expressions completely mediated the relationship between the degree of improvement in irritability and reactive aggression.

## 4. Discussion

In this study, we aimed to investigate the impact of intranasal oxytocin administration on the interaction between irritability and reactive aggression and its target mechanism at the neural level by implementing the facial expression task under functional MRI (fMRI). There are three main findings. First, youth with irritability who received intranasal OXT showed significant decreases in irritability and reactive aggression, compared to the youth who received the placebo (PBO). Second, the degree of improvement in irritability was significantly correlated with the degree of improvement in reactive aggression. Third, this correlation was completely mediated by the increase in the BOLD responses in the superior prefrontal cortex in response to negative and positive facial expressions.

The right superior frontal cortex exhibited group-by-condition effects, such that participants in the oxytocin group exhibited greater increases in neural activity in response to both negative and positive faces compared to changes in the placebo group. The prefrontal cortex is critical for the top-down regulation of affective and cognitive processes, and dysfunction of this region has been linked extensively across mental health disorders [25,26,43,44]. Specifically, youth with high levels of irritability often exhibit decreased activation in prefrontal cortices as well as decreased amygdala–prefrontal connectivity during tasks requiring social evaluation or facial expression processing [45,46]. Existing work has identified increased prefrontal cortical activity following oxytocin administration, with some demonstrating concomitant improvements in social function [47,48,49]. A relatively short-term administration of oxytocin across a 3-week period was sufficient to increase activity in this region, which may reflect the increased engagement of prefrontal regulatory mechanisms that are otherwise utilized to a lesser extent in youth with high irritability, leading to a greater modulation of reactivity. Thus, our findings are consistent with the existing literature and highlight the effectiveness of oxytocin on modulating brain function in a pediatric sample with severe irritability.

We observed alterations in the superior frontal cortex activity that mediated the correlation of an improvement between irritability and reactive aggression. Similarly, previous work demonstrated that the neural responses in the amygdala also mediated a callous–unemotional trait and proactive aggression [50]. Again, we do not attempt to oversimplify the neural pathways contributing to the complex relationships between these psychopathologies; rather, we aim to provide one insightful observation that may serve as a basis for future studies. In this regard, in our previous report using the Affective Stroop task to identify interactions between emotion-responding areas and top-down attention control areas, we observed the engagement of various neural areas (including the anterior cingulate cortex and dorsomedial prefrontal cortex (dm-PFC)) that were correlated with symptom improvement [30]. Thus, this complementary study provides greater detail on the various perspectives and components of the intranasal oxytocin administration on differentiated psychopathologies and their relations, as well as the engagement of neural areas implicated in various underlying neurobiological mechanisms.

Our mediation analyses provide additional clarity regarding the relationship between oxytocin-related neural changes and its role in mediating behavioral improvements, specifically between irritability and reactive aggression. The BOLD responses in the prefrontal cortex fully mediated improvements in response to both positive and negative valence faces, suggesting a shared neural mechanism that underlies reductions in irritability and reactive aggression, consistent with the theories that irritability may be a precursor to reactive aggression. Indeed, previous neuroimaging studies have demonstrated that individuals with lesions on the prefrontal cortex or reduced prefrontal activity exhibit higher levels of reactive aggression [51,52,53]. Our results suggest that the effects of oxytocin may not be limited to reducing threat-related hyperreactivity; rather, it may enhance the salience or cognitive processing of emotionally meaningful stimuli more broadly, allowing youth to better interpret, process, and regulate neural and behavioral responses to a range of socioemotional cues. Importantly, the literature is relatively heterogeneous on the impact of oxytocin, with some studies reporting increased aggression in adults and the improved recognition of happy faces, but not other emotional faces, though this work was conducted in adults [54,55]. Interestingly, these findings indicate significant improvements in self-reported reactive aggression, but not in parent-reported scores. Within the developmental literature, discrepancies in reports from youth and parents are relatively unsurprising considering significant variability in the relationship dynamics, developmental differences in self-perception, and documented differences that are relevant particularly in participants with mental health disorders [56]. Of note, while the difference in parent scores pre- and post-intervention did not achieve statistical significance in this sample, these differences may still be meaningful and obscured by the small sample in this study. While this is a relatively nascent area of research that will require larger samples to disambiguate these nuances, these findings suggest that youth with severe levels of irritability, who are at the highest risk of reactive aggression and subsequent adverse outcomes, may benefit from oxytocin-mediated alterations in neural and behavioral outcomes. Additionally, the specificity of these findings to reactive, but not proactive, aggression highlights the importance of a dimensional model to psychiatry, particularly during childhood and adolescence [57,58,59]. Oxytocin may be a useful therapeutic tool in youth with high levels of irritability and reactive aggression, but may not be as impactful in mitigating proactive aggression.

The majority of the extant literature has examined the impact of a single bolus of oxytocin on neural and behavioral outcomes. Recent findings indicate that repeated administrations of oxytocin have differential effects from single doses of oxytocin in animal and human subjects research, with variable levels of transcriptional changes and fMRI activity [60]. Importantly, while oxytocin has immediate effects by multiple mechanisms including neurotransmitter modulation, it also has long-term neurobehavioral effects via downstream transcriptional modulation [60]. Future investigations would benefit from increased longitudinal sampling, both within the oxytocin administration period and following oxytocin termination. Further, existing therapies for irritability and aggression, including atypical antipsychotics, mood stabilizers, and cognitive behavioral therapy, may be particularly effective when used in conjunction with oxytocin. These therapies likely impact overlapping but partially distinct mechanisms contributing to aggression and irritability, including the neurobiological underpinnings of social cognition and negative thought patterns from lived experiences.

To our knowledge, this is the first study to demonstrate that intranasal oxytocin administration can induce improvements in irritability and aggressive behavior through a neural mechanism. In this regard, it should be noted that there are studies showing increases in aggressive behavior after intranasal oxytocin administration in pediatric populations, but mainly in the context of the presence of participants with a diagnosis of ASD [61]. Thus, future studies are warranted to comprehensively understand the impact of intranasal oxytocin on aggressive behavior related to various psychopathologies in youth, including disruptive behavior and mood disorders and ASD simultaneously. In addition, although the link between proactive aggression and callous–unemotional traits is relatively straightforward and clear in pediatric populations [62], the relationship between irritability and different types of aggressive behavior is more nuanced and complex [63]. It is possible that our study was able to capture only one component of this complex relationship, and the lack of correlation or improvement in the other psychopathologies (including callous–unemotional traits and proactive aggression) might be due to a type II error. Future studies with larger sample sizes are warranted and may address these potential shortcomings of the present analyses.

This study provides critical preliminary evidence of the neural mechanisms linking decreases in irritability to decreases in reactive aggression. Despite the many strengths of this study, there are a few limitations that warrant consideration. The sample size, while comparable to the other fMRI studies in clinical studies of pharmacologic agents with potential therapeutic utility, was small and may be limited in generalizability, particularly to youth with lower levels of irritability or youth with a diagnosis of ASD. The sample size of this study may have been underpowered to detect smaller, but clinically meaningful effects of oxytocin administration (e.g., Type II errors with null findings in parent reported measures). Further, while there were no significant sex differences by group, the overall sample was largely male and caution is warranted in generalizing these results to broader clinical samples. Given that significant sex differences in psychopathology and pharmacological sensitivity have been identified, future work should further disambiguate potential sex differences in oxytocin responsivity. In addition, we also did not collect the menstrual cycle as a covariate for this study, and future studies should consider this to thoroughly examine the impact of intranasal oxytocin in female participants [64]. There are significant genetic differences in the susceptibility and distribution of oxytocin receptors that may contribute to variability in the response profile, as well as demographic features and risk/protection that may render some individuals more susceptible to oxytocin-related changes. Such investigations should be conducted in future work, in conjunction with larger samples to replicate the findings in this study and to assess generalizability.

Taken together, this study provides compelling evidence that intranasal oxytocin use over a relatively short 3-week intervention period modulates the prefrontal regulation of neural responses supporting facial expression processing in youth with high levels of irritability. These alterations are directly linked with clinically meaningful reductions, specifically in reactive, but not proactive, aggression. These findings add to the burgeoning literature supporting oxytocin as a potential therapeutic intervention for irritability and subsequently reactive aggression, which have significant associations with adverse long-term outcomes.

## Figures and Tables

**Table 1:** Participant demographics.

	Youth with Disruptive Behavior Disorders on OXT Treatment (n = 21)	Youth with Disruptive Behavior Disorders on PLB Treatment (n = 19)	*p* Value
Age	14.4 (2.1)	14.2 (2.2)	0.85
Sex	8 girls/13 boys	3 girls/18 boys	0.09
IQ	103.4 (16.2)	100.7 (14.6)	0.91
Diagnosis (Primary)			
ADHD	12 (57.1%)	10 (52.6%)	
DMDD	4 (19.0%)	4 (21.1%)	
CD	2 (9.5%)	0 (0%)	
Other	2 (9.5%)	5 (26.3%)	

Other: Mood Disorder, not otherwise specified; Bipolar Disorder, not otherwise specified; Anxiety Disorder, not otherwise specified; Generalized Anxiety Disorder; Social Phobia; Panic Disorder; Social Anxiety Disorder.

**Table 2:** Symptom profiles in baseline and follow-up.

	Youth with DBDs on OXT Treatment (n = 21)		Youth with DBDs on PLB Treatment (n = 19)		$p\;\mathrm{Value}/\eta_{p}^{2}$
	Baseline	Follow-Up	Baseline	Follow-Up	
ARI-Y	6.71 (2.15)	3.57 (2.36)	6.16 (2.95)	6.16 (3.15)	<0.001 */0.363
ARI-P	7.76 (2.81)	4.14 (2.82)	8.53 (2.09)	6.31 (3.04)	0.20/0.043
RPAQ-Y	15.14 (6.09)	8.43 (6.70)	16.35 (7.89)	14.09 (6.72	0.07/0.177
RA-Y	12.33 (3.85)	6.95 (4.76)	12.37 (5.20)	10.95 (4.65)	0.005 */0.190
PA-Y	2.80 (2.84)	1.55 (2.66)	3.96 (2.95)	3.13 (3.78)	0.54/0.010
RPAQ-P	19.71 (7.27)	10.86 (6.24)	22.42 (6.53)	16.82 (10.22)	0.21/0.041
RA-P	14.86 (3.92)	8.81 (4.26)	16.05 (3.87)	12.40 (6.28)	0.11/0.066
PA-P	2.81 (2.84)	1.55 (2.66)	3.96 (2.95)	3.13 (3.78)	0.54/0.010

Abbreviations: DBDs; Disruptive Behavior Disorders; ARI-Y: Youth-reported Affective Reactivity Index; ARI-P: Parent-reported Affective Reactivity Index; ICU-Y: Youth-reported Inventory of Callous–Unemotional Trait; ICU-P: Parent-reported Inventory of Callous–Unemotional Trait; RPAQ-Y: Youth-reported Reactive–Proactive Aggression Questionnaire; RA-Y: Youth-reported Reactive Aggression; PA-Y: Youth-reported Proactive Aggression; RPAQ-P: Parent-reported Reactive–Proactive Aggression Questionnaire; RA-P: Parent-reported Reactive Aggression; PA-P: Parent-reported Proactive Aggression. ( ) = Standard Deviation. *: *p* < 0.05.

**Table 3:** Brain regions showing significant interactions.

	Coordinates of Peak Activation ^b^							
Region ^a^	Left/Right	BA	x	y	z	F	Voxels	η^2^
Group by time								
Superior Frontal Cortex	Right	8	16.5	28.5	50.5	22.63	40	1.305

^a^ According to the Talairach Daemon Atlas (http://www.nitc.org/projects/tal-daemon/). ^b^ Based on the Tournoux and Talairach standard brain template.

## Data Availability

Data supporting the findings of this study are available from the corresponding author (Soonjo Hwang) upon request.
